# Gut Microbiota and Hepatocellular Carcinoma: Metabolic Products and Immunotherapy Modulation

**DOI:** 10.1002/cam4.70914

**Published:** 2025-05-02

**Authors:** Kunmin Xiao, Kexin Li, Kunlin Xiao, Jinzu Yang, Lei Zhou

**Affiliations:** ^1^ Department of Oncology Longhua Hospital, Shanghai University of Traditional Chinese Medicine Shanghai China; ^2^ Department of Traditional Chinese Medicine Peking Union Medical College Hospital, Peking Union Medical College, Chinese Academy of Medical Science Beijing China; ^3^ Department of Emergency Zhongshan Hospital, Fudan University Shanghai China

**Keywords:** gut microbiota, hepatocellular carcinoma, immunotherapy, metabolites

## Abstract

**Background:**

The relationship between hepatocellular carcinoma (HCC) and gut microbiota has gained attention for its impact on HCC immunotherapy.

**Methods:**

Key gut microbial metabolites, including bile acids, toll‐like receptor 4, short‐chain fatty acids, and bacterial toxins, contribute to HCC progression and influence immune responses through the gut‐liver axis. As immune checkpoint inhibitors (ICIs) become common in HCC treatment, modulating the gut microbiota offers new strategies to enhance ICIs efficacy. However, individual differences in microbial composition introduce challenges, with some HCC patients showing resistance to ICIs.

**Results:**

This review summarizes the latest findings on the role of gut microbiota in HCC and explores emerging therapeutic approaches, including fecal microbiota transplantation, probiotics, antibiotics, and natural compounds.

**Conclusions:**

The focus is on translating these insights into personalized medicine to optimize ICIs responses and improve HCC treatment outcomes.

## 1 Background

Primary liver cancer remains one of the most aggressive malignancies, with high incidence and mortality rates worldwide. In 2022, there were 865,269 new cases of liver cancer globally, and 757,948 deaths, making it the 6th most common malignant tumor worldwide and the 3rd leading cause of cancer‐related deaths; approximately 80% of these cases are hepatocellular carcinoma (HCC) [1]. HCC develops through multifactorial processes, with established risk factors including chronic hepatitis B (HBV) and C infections (HCV), cirrhosis, and non‐alcoholic fatty liver disease (NAFLD) [2]. However, the precise molecular mechanisms driving HCC progression require further elucidation.

Recent research has elucidated the pivotal role of gut microbiotas in HCC development [3]. The gut microbiota exists stably under the combined effects of mucosal tissue and the immune system [4]. The gut microbiota, a complex and diverse community of microorganisms residing within the intestinal mucosa, plays a crucial role in maintaining immune homeostasis and promoting metabolic health. The composition of the gut microbiota is influenced by various factors, such as external environmental factors, dietary habits, and the immune system, all of which can alter microbial diversity and functionality [5]. To characterize this complex ecosystem, researchers have developed a variety of advanced detection technologies. These include traditional culture‐dependent microbial techniques, 16S rRNA gene sequencing analysis for identifying and quantifying bacterial species in the gut [6], and metagenomic sequencing, which offers a comprehensive view of the genetic composition and functional potential of the gut microbial community [7]. Metabolomic approaches, such as liquid chromatography‐mass spectrometry, further enable the detection and quantification of microbial metabolites linked to HCC [8]. Emerging non‐invasive techniques, particularly volatile organic compound analysis from breath tests, are providing novel insights into gut‐liver interactions and their potential role in HCC development [9].

While early findings highlight the importance of the gut microbiota in modulating immune responses and influencing HCC outcomes, the mechanisms linking microbial dysbiosis to HCC remain underexplored. Further research is necessary to elucidate how gut microbiota‐derived metabolites and immune regulatory functions contribute to HCC pathogenesis, potentially opening new avenues for prevention and treatment.

## 2 Gut‐Liver Axis and Intestinal Mucosal Barrier: The Anatomical Link Between Gut Microbiota and HCC

The “gut‐liver axis” concept, introduced by Marshall in 1998, describes the bidirectional communication between the gastrointestinal tract and the liver via the portal circulation [10]. The liver receives approximately 75% of its blood supply from the portal vein, transporting nutrients, microbial metabolites, and bacterial products from the gut. This close anatomical relationship makes the liver highly vulnerable to gut‐derived toxins and inflammatory signals, especially when the intestinal barrier is compromised. In turn, the liver affects the gut by secreting bile acids (BAs) and other factors, influencing the gut microbiota and its function [11]. This bidirectional interaction, known as the gut‐liver axis, forms the anatomical and functional basis for the involvement of gut microbiota in HCC. Similar to the gut‐liver axis, other microbial axes, such as the oral‐gut axis in breast cancer and gynecological cancers, have been shown to play critical roles in cancer development and progression. These axes highlight the systemic impact of microbial communities on tumorigenesis, immune regulation, and treatment response, further underscoring the importance of microbial interactions in cancer biology [12, 13].

The intestinal mucosal barrier is a key element of the gut‐liver axis, responsible for separating the body from gut microbes and harmful substances. This barrier consists of the mechanical barrier, the functional barrier, and the immune barrier. Together, they maintain intestinal integrity and prevent microbial translocation into the bloodstream [14]. When this barrier is disrupted, the permeability of the intestinal epithelium increases, allowing microbial products, such as lipopolysaccharides (LPS), to enter the portal circulation. LPS activates toll‐like receptor 4 (TLR4) on liver Kupffer cells, triggering inflammatory signaling pathways such as NF‐κB, which promote hepatic inflammation, fibrosis, and carcinogenesis [15].

Dysbiosis, an imbalance in the gut microbiota, further exacerbates intestinal permeability by impairing tight junction proteins in the epithelium, creating a “leaky gut” [16]. This leads to an influx of microbial‐derived products that overwhelm the liver's detoxification capacity, driving chronic inflammation. Persistent liver inflammation contributes to a pro‐tumorigenic environment by promoting immune cell dysfunction, fibrosis, and hepatocyte injury. Over time, this cycle of inflammation and tissue repair creates conditions conducive to the development of HCC [17]. Thus, the disruption of the gut‐liver axis and the intestinal mucosal barrier plays a critical role in the pathogenesis of HCC.

## 3 Intestinal Microbiota Promotes HCC Through Metabolic Products

**FIGURE 1:**
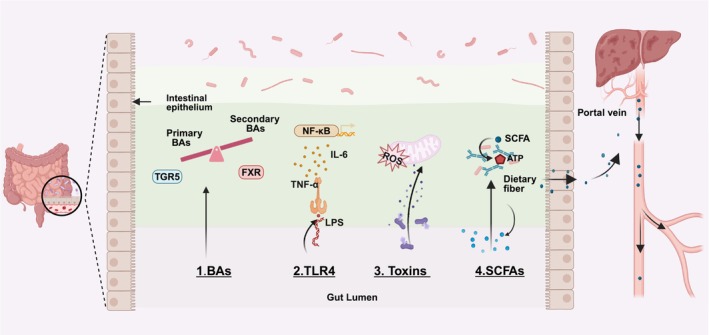
Mechanisms by which gut microbiota metabolites promote HCC development. BAs bind to their receptors FXR and TGR5, regulating BAs metabolism, while LPS activate TLR4 and induce pro‐inflammatory cytokines through the NF‐κB pathway. Toxins promote oxidative stress and cellular damage, while SCFAs affect energy metabolism. Together, these metabolites create a tumor‐promoting microenvironment in the liver. ATP: adenosine triphosphate; BAs: bile acids; FXR: farnesoid X receptor; LPS: lipopolysaccharide; ROS: reactive oxygen species; SCFAs: short‐chain fatty acids; TGR5: Takeda G protein‐coupled receptor 5; TLR4: toll‐like receptor 4.

The interaction between the gut microbiota and HCC is primarily mediated by its metabolic products, which include alterations in BAs metabolism, activation of TLR4, production of toxins, and regulation of short‐chain fatty acids (SCFAs) levels. These mechanisms not only elucidate the complex relationship between the gut microbiome and the liver but also present new opportunities for HCC treatment (Figure 1).

### 3.1 Bile Acids

The liver is the primary site for the production of BAs, which play a crucial role not only in the digestive process but also in liver health and the occurrence of HCC. BAs can be temporarily stored in the gallbladder and released into the intestine as needed to aid in the digestion of fats and the absorption of fat‐soluble vitamins. In the small intestine, BAs are reabsorbed and recirculated through the liver, a process that is essential for maintaining the homeostasis of the gut‐liver axis. The gut microbiota is involved in multiple stages of BAs metabolism, including the synthesis, conjugation, reabsorption, deconjugation, and conversion of primary BAs to secondary BAs. Disruptions in this metabolic process can lead to alterations in the composition and abundance of BAs, thereby disrupting homeostasis and affecting liver health [18].

Certain bacteria, such as Clostridium species, exert pro‐carcinogenic effects by converting primary BAs into deoxycholic acid (DCA). DCA can induce deoxyribonucleic acid (DNA) damage and promote HCC progression by SASP‐associated tumor‐promoting factors [19]. On the other hand, bacteria that are rich in bile salt hydrolase (BSH), such as Bifidobacterium and Lactobacillus species, can transform primary BAs like chenodeoxycholic acid and cholic acid into secondary BAs, including lithocholic acid, DCA, and ursodeoxycholic acid. This conversion occurs through a two‐step process: initial deconjugation by BSH, followed by further modifications mediated by enzymes such as 7‐alpha‐dehydroxylase or 7‐alpha‐hydroxysteroid dehydrogenase [20, 21]. Studies have shown that vancomycin treatment reduces the population of BSH‐producing bacteria, decreases the proportion of secondary BAs, and consequently accelerates the growth of HCC xenograft tumors. This suggests that secondary BAs may play a crucial role in inhibiting HCC progression [22].

BAs receptors, such as Farnesoid X receptor (FXR) and Takeda G protein‐coupled receptor 5 (TGR5), play crucial roles in the interaction between BAs metabolism and the gut microbiota. FXR maintains BAs homeostasis and regulates liver health by inhibiting BAs synthesis. In patients with non‐alcoholic steatohepatitis (NASH)‐related HCC, abnormal elevations in fibroblast growth factor 19 (FGF19) levels are closely associated with BAs metabolism dysregulation [23]. In HCC, FGF19 overexpression promotes tumor progression and poorer prognosis by activating the Wnt/β‐catenin signaling pathway, which drives cell proliferation and survival. Targeting the FGF19‐FGFR4 axis has emerged as a promising therapeutic strategy. FGF19 analogs, designed to mimic the natural ligand while avoiding its tumor‐promoting effects, have demonstrated anti‐fibrotic and anti‐tumor properties by suppressing BAs synthesis and modulating downstream signaling pathways. For example, FGF401, a selective FGF19‐FGFR4 signaling inhibitor, has shown favorable safety profiles and preliminary clinical efficacy in FGFR4/KLB‐positive HCC patients. In early‐phase clinical trials, FGF401 exhibited promising anti‐tumor activity both as a monotherapy and in combination with spartalizumab, a programmed death‐1 (PD‐1) inhibitor. However, further studies are needed to validate its long‐term efficacy and address potential resistance mechanisms [24].

Given the close relationship between the gut microbiota and BAs metabolism, modulating the gut microbiota to improve BAs metabolism has emerged as a novel strategy for treating HCC. For instance, XYXD increases the abundance of Bacteroides and Lactobacillus, which promote the production of BSH. BSH converts conjugated BAs into primary BAs while reducing the abundance of Eubacterium, a key bacterium involved in the conversion of primary BAs to secondary BAs. This shift increases the levels of primary BAs, which trigger NKT cells in the liver to produce interferon‐γ (IFN‐γ), thereby exerting anti‐HCC immune effects [25]. Additionally, studies have demonstrated that specific natural compounds, such as chlorogenic acid and caffeic acid, can regulate the gut microbiota (e.g., Ruminococcaceae UCG‐004, Lachnospiraceae, and Prevotellaceae 9), further improving BAs metabolism and inhibiting HCC progression [26, 27]. These findings offer new insights and potential therapeutic targets for treating HCC by modulating the gut microbiota and BAs metabolism.

### 3.2 Toll‐Like Receptor 4

Toll‐like receptors (TLRs) play a crucial role in the initiation and progression of HCC by recognizing pathogen‐associated molecular patterns (PAMPs)and damage‐associated molecular patterns. The gut microbiota influences the activity of TLRs and intestinal barrier function, thereby modulating HCC development. LPS, a key ligand for TLR4, is produced by the gut microbiota. Dysbiosis of the gut microbiota can increase the translocation of LPS and other molecules to the liver, subsequently activating TLR4 [28].

TLR4 is widely expressed in hepatocytes, hepatic stellate cells, and Kupffer cells. Upon binding with LPS, TLR4 activates the NF‐κB and JNK/MAPK signaling pathways, inducing the release of pro‐inflammatory cytokines such as interleukin‐6 (IL‐6) and tumor necrosis factor‐α (TNF‐α), which promote hepatocyte proliferation, survival, and epithelial‐mesenchymal transition (EMT), thereby enhancing the invasiveness and metastatic potential of HCC [29, 30]. Additionally, LPS‐TLR4 signaling promotes the differentiation of hepatic progenitor cells into myofibroblasts, leading to aberrant tumor signaling and accelerating HCC development [31].

TLR4‐mediated inflammation plays a critical role in the progression of HCC, with the gut microbiota influencing its activity by regulating TLR4 ligands such as LPS. Studies have shown that in PtenΔhep mice, relying on the TLR4 rather than TLR2 signaling pathway promotes the development of HCC by exacerbating liver inflammation, producing more IL‐6 and TNF‐α. Antibiotic treatment can reduce LPS levels in the portal vein and inhibit tumor growth, demonstrating that the gut microbiota is a key source of TLR4 ligands [32]. TLR4 ablation sensitized the liver to carcinogen‐induced toxicity by blocking NF‐κB activation and increasing susceptibility to reactive oxygen species (ROS)‐induced damage, while reducing inflammation‐mediated compensatory proliferation. Reconstitution of TLR4‐expressing myeloid cells in TLR4‐deficient mice restored diethylnitrosamine (DEN)‐induced hepatic inflammation and proliferation, indicating a paracrine mechanism of LPS in tumor promotion [33]. In contrast to traditional studies focusing on bone marrow‐derived inflammatory pathways, research by Dapito et al. [34] revealed that non‐bone marrow‐derived hepatic stellate cells and hepatocytes are the primary targets of LPS. Dysbiosis of the gut microbiota promotes DEN‐induced HCC in mice by activating TLR4 and accelerates compensatory proliferation following hepatocyte injury through its effects on hepatocytes and hepatic stellate cells. While TLR4 and the gut microbiota are not initiating factors in HCC, they play a crucial role in tumor progression by promoting hepatocyte proliferation and inhibiting apoptosis. Studies have also shown that TLR4 gene inactivation or gut sterilization can reduce HCC incidence by approximately 80%, whereas long‐term treatment with low‐dose LPS significantly accelerates HCC progression. Even in advanced HCC, antibiotic‐induced gut sterilization can effectively inhibit tumor growth, offering a potential strategy for HCC prevention in patients with chronic liver injury.

### 3.3 Toxins

Bacterial toxins produced by the gut microbiota can damage host DNA through various mechanisms, potentially leading to carcinogenesis. These toxins may induce DNA damage indirectly via reactive oxygen species or act directly on host DNA. When the extent of DNA damage surpasses the cell's repair capacity, carcinogenesis may occur. Aflatoxin, a well‐known potent carcinogen, not only directly alters the composition of the gut microbiota but is also closely associated with the development of HCC [35].

In addition to bacterial toxins, toxic trace elements such as arsenic have been implicated in increasing HCC risk by disrupting the gut microbiota. Once arsenic enters cells, it is detoxified and expelled by glutathione, a key antioxidant. Higher cellular glutathione levels are associated with lower intracellular arsenic concentrations, highlighting the role of glutathione in mitigating arsenic‐induced toxicity. However, chronic arsenic exposure can overwhelm this detoxification mechanism, leading to gut microbiota dysbiosis and subsequent liver damage, which may contribute to HCC development [36]. Studies have shown that arsenic exposure elevates the abundance of Gram‐negative bacteria in the gut, increases intestinal permeability, and facilitates the translocation of LPS. Through systemic circulation, LPS can reach the liver, triggering inflammation and promoting the development of HCC. In arsenic‐induced liver cancer models, germ‐free mice, compared to conventional mice, excrete higher levels of arsenic and its metabolites in urine, with an elevated ratio of monomethylarsonic acid to dimethylarsinic acid, while the concentration of arsenic in feces is lower. Further experimental models revealed that the gut microbiota is involved in arsenic absorption, whereas germ‐free mice exhibit downregulation of enzymes related to arsenic methylation [37].

Further research has shown that the depletion of gut microbiota enhances arsenic toxicity in the liver by altering the expression of genes related to the p53 pathway, as well as other liver cancer‐associated genes. This increases the risk of HCC in germ‐free mice. These findings underscore the potential role of the gut microbiota in HCC development, revealing the complex interplay between the microbiota and environmental toxins in promoting liver carcinogenesis [38].

### 3.4 Short‐Chain Fatty Acids

SCFAs are substances produced by the fermentation of various indigestible polysaccharides (e.g., cellulose and low‐digestibility sugars) by gut microbiota, which can be absorbed by the intestine. SCFAs consist of carbon chains with 1 to 6 atoms, primarily including acetate, propionate, and butyrate. Acetate and propionate are mainly produced by Bacteroidetes, while butyrate is primarily provided by Firmicutes [39]. SCFAs serve as a direct energy source for intestinal mucosal cells, reducing apoptosis and maintaining the integrity of the mechanical barrier. Additionally, SCFAs lower intestinal PH, inhibiting the growth and colonization of pathogens and alleviating inflammatory responses. When SCFAs production is disrupted, it may lead to increased intestinal barrier permeability, excessive absorption of bacterial metabolites, and intestinal endotoxemia, subsequently activating the LPS‐TLR4 pathway and causing hepatocellular damage [40].

SCFAs, as metabolic products of the gut microbiota, have been found to be associated with the development of HCC. HBx, an oncogenic protein encoded by HBV, is closely linked to the pathogenesis of HCC. Studies have shown that SCFA intake promotes the expression of suppressor of cytokine signaling 2 in HBx transgenic mice and downregulates various inflammatory signaling pathways, including Ras, PI3K, VEGF, fibroblast growth factor, and epidermal growth factor, thereby slowing the progression of HCC [41]. Clinical investigations by Behary et al. demonstrated significant changes in the structure and function of the gut microbiota in patients with NAFLD‐HCC, including an increase in bacteria such as *Bacteroides xylanisolvens* , *Ruminococcus gnavus* , and *Clostridium bolteae* , as well as a notable rise in SCFA‐producing bacteria. Furthermore, levels of SCFAs and their derivatives were found to be significantly elevated in both the feces and serum of NAFLD‐HCC patients. These SCFAs can trigger immune responses by promoting the expansion of effector IL‐10^+^Tregs, reducing the proliferation of cytotoxic CD8^+^T cells, and suppressing the body's antigenic response, ultimately leading to immune suppression [42].

### 3.5 Dietary Interventions and Microbial Metabolites in HCC

Mounting evidence underscores the pivotal role of dietary factors in shaping gut microbial composition and metabolic output, thereby influencing HCC pathogenesis and clinical outcomes. Clinical evidence has shown that specific dietary patterns, including high‐fiber diets [43], western‐style diets [44], the ketogenic diet [45] and the Mediterranean diet, are associated with prognosis in HCC patients [46, 47]. Adherence to a Mediterranean diet has been linked to reduced liver‐related mortality and improved overall survival, potentially due to its anti‐inflammatory and antioxidant properties [48, 49, 50, 51]. Likewise, high‐fiber diets have been associated with better treatment tolerance and reduced disease progression in HCC cohorts [43]. However, these clinical studies have not directly investigated the role of gut microbiota in mediating these dietary effects, highlighting the need for mechanistic insights from preclinical models.

Preclinical studies in murine models provide mechanistic evidence linking dietary interventions to gut microbiota modulation and HCC progression. High‐fiber diets enrich Bifidobacterium species and increase SCFA production, enhancing intestinal barrier function, reducing systemic inflammation, and inhibiting tumor growth [52]. Conversely, high‐fat diets, such as the steatohepatitis‐inducing STHD‐01 and high‐fat/high‐cholesterol diets, promote dysbiosis and hepatocarcinogenesis through harmful metabolites like secondary BAs. Specifically, these diets upregulate BAs synthesis enzymes (e.g., cytochrome P450 cholesterol 7α‐hydroxylase) and alter gut microbiota composition (e.g., increased Mucispirillum and Desulfovibrionaceae, decreased Bacteroides and Bifidobacterium). These alterations lead to impaired BAs metabolism, elevated LPS levels, insulin resistance, and oxidative stress, mediated by hepatic ROS accumulation and pro‐inflammatory immune responses. Together, these mechanisms establish a tumor‐promoting microenvironment that drives HCC progression [53, 54].

Although direct evidence linking dietary interventions to enhanced efficacy of immune checkpoint inhibitors (ICIs) in HCC is limited, the modulation of gut microbiota and its metabolites by diet provides a plausible mechanism through which dietary strategies could indirectly influence HCC progression. Future research should explore the potential of combining dietary interventions with other microbiota‐targeted therapies to optimize HCC treatment outcomes.

## 4 Impact of Gut Microbiota on HCC via Immune Microenvironment Modulation

**FIGURE 2:**
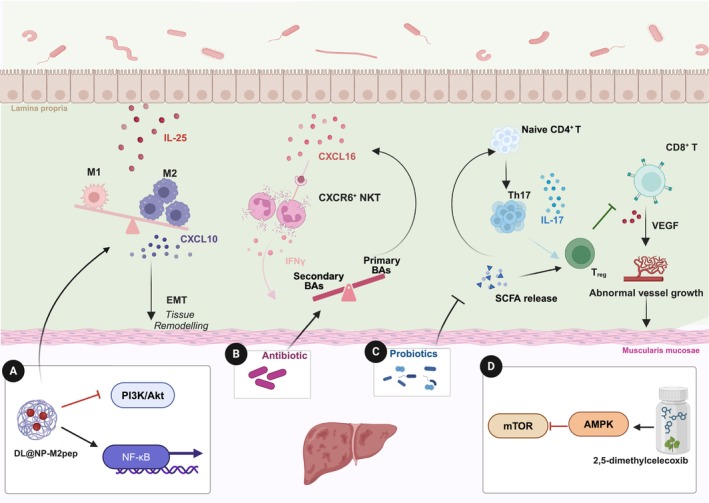
Gut microbiota influence on immune modulation in HCC. The gut microbiota and its metabolites impact various immune cell types and pathways, affecting tumor progression and responses to therapies. (A) DL@NP‐M2pep promotes M2‐to‐M1 macrophage reprogramming by inhibiting the PI3K/Akt pathway and activating NF‐κB signaling; (B) Antibiotics modulate BAs metabolism, influencing NKT cell accumulation and enhancing their antitumor activity; (C) Probiotics modulate SCFAs levels to suppress IL‐17‐producing Th17 cells, promote regulatory T cells, and inhibit tumor growth in HCC by altering immune responses; (D) The gut microbiota regulates angiogenesis by modulating SCFAs levels and CD8^+^T cell function, while pharmacological agents like the celecoxib derivative 2,5‐dimethyl celecoxib enhance CD8^+^T cell cytotoxicity and inhibit tumor growth in HCC by targeting the gut microbiota and the AMPK‐mTOR pathway. BAs: bile acids; CXCL10: C‐X‐C motif chemokine ligand 10; CXCL16: C‐X‐C motif chemokine ligand 16; CXCR6^+^ NKT: C‐X‐C chemokine receptor type 6 positive natural killer T cells; EMT: epithelial‐mesenchymal transition; IFN‐γ: interferon‐γ; IL‐17: interleukin‐17; IL‐25: interleukin‐25; M1/M2: macrophage type 1/type 2; PI3K/Akt: phosphatidylinositol 3‐kinase/protein kinase B; SCFA: short‐chain fatty acid; Th17: T helper type 17 cells; Treg: regulatory T cell; VEGF: vascular endothelial growth factor.

The HCC immune microenvironment is a complex network composed of tumor cells, immune cells, stromal cells, endothelial cells, and pericytes. These cells produce non‐cellular components, including inflammation‐related cytokines, growth factors, and extracellular matrix proteins [55]. Key features of the immune microenvironment in HCC include dendritic cell dysfunction, NK cell impairment, T cell exhaustion, and the infiltration of immunosuppressive cells, all of which collectively promote tumor growth [56]. The gut microbiota, through its metabolic products and immunomodulatory functions, profoundly influences the activity and function of immune cells within the liver, thereby exerting a significant impact on the HCC immune microenvironment (Figure 2).

### 4.1 Tumor‐Associated Macrophages

Tumor‐associated macrophages (TAMs) consist of peripheral blood monocyte‐derived macrophages and liver‐resident Kupffer cells, known for their high plasticity, which allows them to switch between anti‐tumor macrophage type 1 (M1) and pro‐tumor macrophage type 2 (M2) phenotypes [57]. In HCC, TAMs predominantly adopt the M2 phenotype, which facilitates immune evasion, promotes T cell exhaustion, and correlates with poor patient prognosis. M2 TAMs contribute to HCC progression by secreting immunosuppressive cytokines such as interleukin‐10 (IL‐10) and transforming growth factor‐β (TGF‐β), promoting angiogenesis, and recruiting regulatory T cells (Tregs). Furthermore, TAMs express elevated levels of PD‐L1, diminishing the cytotoxic functions of CD8^+^T cells, a phenomenon exacerbated by IL‐6 and HCC‐derived exosomes upregulating PD‐L1 in TAMs [58, 59]. Thus, reprogramming TAMs from M2 to M1 is critical for effective cancer immunotherapy.

Gut microbiota metabolites, particularly LPS, SCFAs, BAs, and tryptophan metabolites, play a significant role in regulating TAMs polarization. PAMPs, such as LPS and bacterial DNA, are key mediators of this process. LPS, a component of the outer membrane of Gram‐negative bacteria, activates monocyte‐derived macrophages through the TLR4/NF‐κB pathway, driving M1 polarization and promoting the production of pro‐inflammatory cytokines [60]. Similarly, bacterial DNA, which contains unmethylated CpG motifs, activates TLR9 signaling in macrophages, leading to the secretion of pro‐inflammatory cytokines and the recruitment of immune cells to the tumor site [61]. In contrast, SCFAs like butyrate favor M2 polarization by increasing IL‐10 levels, inhibiting histone deacetylase 1, and promoting histone acetylation [62]. Moreover, secondary BAs (e.g., DCA) and indole‐3‐acetic acid from dysbiotic gut microbiota further drive M2 polarization through specific signaling pathways, contributing to an immunosuppressive tumor microenvironment (TME) [63, 64]. Additionally, tryptophan metabolites, such as indole and kynurenine, regulate TAMs polarization and T cell function through the aryl hydrocarbon receptor (AhR) [65]. The absence of AhR leads to inflammatory polarization of TAMs and increased infiltration of CD8^+^T cells in the TME. Studies have shown that dietary tryptophan depletion reduces AhR activity in TAMs and promotes the accumulation of TNFα^+^IFNγ^+^ CD8^+^ T cells within tumors, thereby enhancing anti‐tumor immune responses [64].

In HCC patients, particularly those with microvascular invasion, the gut microbiota exhibits significant alterations, with increased abundance of Klebsiella and Proteobacteria and reduced levels of beneficial Firmicutes and Ruminococcaceae. This dysbiosis is associated with enhanced M2 polarization, which promotes HCC invasion and metastasis through the secretion of chemokines like C‐X‐C motif chemokine ligand 10 (CXCL10) and activation of the EMT pathway [66]. Moreover, interleukin‐25 (IL‐25) has emerged as a critical factor in HCC progression, selectively activating macrophages within the TME to secrete CXCL10, further stimulating the EMT pathway and driving tumor growth and metastasis. Gut microbiota dysbiosis, particularly the increase in Gram‐negative bacteria like Proteobacteria, promotes IL‐25 secretion through the proliferation of tuft cells in the colon [63]. This cascade contributes to M2 macrophage polarization and HCC progression.

Recent research highlights the role of D‐lactate (DL), a gut microbiota metabolite, in reshaping the liver immune microenvironment. DL reaches the liver via the portal vein, enhancing the pathogen‐clearing capacity of Kupffer cells and promoting M2‐to‐M1 conversion of TAMs by inhibiting the PI3K/Akt pathway and activating NF‐κB signaling. A novel nanoparticle formulation (DL@NP‐M2pep) has shown promise in an HCC mouse model, promoting M2‐to‐M1 reprogramming and enhancing the efficacy of ICIs, significantly improving survival in anti‐CD47 antibody therapy [67]. These studies suggest that targeting TAMs reprogramming via gut microbiota modulation may hold potential for improving therapeutic outcomes in HCC patients.

### 4.2 Natural Killer T Cells

Natural killer T cells (NKT) in the human liver account for approximately 25% to 40% of total lymphocytes, with CD56^bright^ and CD56^dim^ NK cells each comprising 50% of the hepatic NKT cell population [68]. The elevated proportion of NKT cells in the liver is closely associated with their critical role in tumor surveillance. NKT cells eliminate virus‐infected and malignant cells, while also inhibiting liver fibrosis by targeting hepatic stellate cells [69]. However, in HCC patients, the frequency of NKT cells within tumor tissues is significantly reduced, accompanied by impaired cytotoxic activity and cytokine production, which correlates with poor clinical outcomes [70]. Butyrate deficiency has been shown to impair interleukin‐18 (IL‐18) production in Kupffer cells and hepatocytes via GPR109A receptor signaling. Disruption of the IL‐18/interleukin‐18 receptor (IL‐18R) axis consequently inhibits mitochondrial function and maturation of liver‐resident NK cells. Notably, dietary supplementation with *Clostridium butyricum* , a butyrate‐producing bacterium, or its clinical administration has been demonstrated to restore the maturation and functionality of liver‐resident NKT cells compromised by early antibiotic exposure [71].

The study by Chi Ma et al. further elucidates the critical role of gut symbiotic bacteria in regulating liver antitumor immunity, particularly through modulating the accumulation of C‐X‐C chemokine receptor type 6 positive natural killer T (CXCR6^+^ NKT) cells in the liver. Their research demonstrates that alterations in the gut microbiota can selectively promote the expansion of NKT cells, which exhibit enhanced activity and increased secretion of IFN‐γ, effectively suppressing tumor growth, regardless of mouse strain, sex, or the presence of liver tumors. This process is regulated by the expression of C‐X‐C motif chemokine ligand 16 (CXCL16), the only ligand for CXCR6, in liver sinusoidal endothelial cells (LSECs) [72]. BAs metabolism plays a crucial role in this mechanism. Primary BAs induce the expression of CXCL16, thereby promoting the accumulation of NKT cells in the liver, while secondary BAs inhibit this process. Antibiotic treatment targeting Gram‐positive bacteria, such as Clostridium species, increases the levels of primary BAs and decreases secondary BAs, thus enhancing the antitumor activity of NKT cells. Further research revealed that secondary BAs suppress the expression of CXCL16 in LSECs, whereas primary BAs promote the accumulation of NKT cells by upregulating CXCL16 expression [73]. These results suggest that gut microbiota, by regulating BAs metabolism, can influence NKT cell accumulation and exert antitumor effects, providing new directions for the development of immunotherapy strategies for HCC.

### 4.3 T Helper 17 Cells

T helper 17 cells (Th17), a subset of CD4^+^T cells, are primarily found in gut‐associated tissues, where they play a dual role in both supporting immune defense and contributing to autoimmune inflammation [74]. Intestinal Th17 cells exist in two forms: one that aids the host immune system, and another that contributes to various autoimmune inflammatory diseases [75]. Their induction and accumulation are highly influenced by specific gut microbiota, particularly through microbial metabolites like BAs and SCFAs. The plasticity of Th17 cells in diseases such as cancer is increasingly linked to gut microbial composition, although the precise mechanisms remain under investigation.

Recent studies have explored how altering the gut microbiota can modulate Th17 cell function, with significant implications for HCC suppression. A notable example is the development of a probiotic mixture, Prohep, composed of *Lactobacillus*, *Escherichia coli* , and inactivated VSL#3 strains [76]. In a mouse model, Prohep significantly reduced tumor size by approximately 40%, primarily by downregulating interleukin‐17‐producing Th17 cells within the tumor. The probiotics not only altered the gut microbiota but also increased anti‐inflammatory metabolites, suppressed Th17 polarization, and promoted the differentiation of regulatory T cells (Treg/Tr1). These changes inhibited tumor angiogenesis and growth, suggesting that modulating the gut microbiota could be a promising approach for influencing Th17‐mediated immune responses in HCC therapy [77].

### 4.4 CD8^+^T

The gut microbiota plays a pivotal role in regulating CD8^+^ T cell activity within the TME, particularly through the production of SCFAs like butyrate. These microbial metabolites influence CD8^+^T cell function by interacting with G‐protein‐coupled receptors, modulating metabolic programming, and enhancing or suppressing cytotoxic activity [78, 79]. In NAFLD‐HCC, SCFAs, particularly butyrate, can promote an immunosuppressive environment by enhancing regulatory T cell (Treg) function and inhibiting CD8^+^T cell cytotoxicity. Furthermore, dysbiosis of gut microbiota alters SCFA levels, leading to compromised gut barrier integrity and the translocation of pro‐inflammatory bacterial metabolites to the liver. These changes exacerbate liver inflammation and cancer progression through activation of Kupffer cells and hepatic stellate cells [42].

In patients with HBV‐HCC, dysbiosis of beneficial gut microbiota, particularly changes in the Firmicutes and Bacteroidetes phyla, negatively regulates liver function and T cell immune responses. This suggests potential avenues for microbiota‐based prevention and intervention strategies in enhancing antitumor immunity for HBV‐HCC [80]. Vagotomy studies have revealed another layer of complexity in the gut‐liver axis, showing that hepatic vagotomy reduces HCC burden by promoting CD8^+^T cell activation. Conversely, stimulation of parasympathetic nerve activity through the vagus nerve releases acetylcholine, which suppresses CD8^+^T cell activity via the CHRM3 receptor, impacting liver immunity [81].

Pharmacological approaches have also demonstrated the ability to modulate gut microbiota and CD8^+^T cell activity. For instance, the celecoxib derivative 2,5‐dimethyl celecoxib inhibits PD‐1 expression and enhances IFN‐γ production in NK and T cells through the gut microbiota‐AMPK‐mTOR pathway, suppressing HCC growth in mouse models [82]. Similarly, sterol interventions such as stigmasterol have been shown to modulate specific Lactobacillus species, leading to increased IFN‐γ^+^CD8^+^T cells and tumor cell apoptosis, providing promising therapeutic avenues for HCC treatment [83].

## 5 Gut Microbiome as a Biomarker for Response to HCC Immunotherapy

ICIs have become a key treatment option for HCC, with the combination of the anti‐PD‐L1 antibody atezolizumab and the anti‐vascular endothelial growth factor antibody bevacizumab now being the preferred first‐line therapy for advanced HCC [84]. However, despite their success, challenges remain, including low response rates, resistance, and adverse effects like hyperprogression. Notably, only 15%–20% of HCC patients respond to PD‐1/PD‐L1 inhibitors, and approximately 25% experience severe immune‐related adverse events [84, 85, 86]. Thus, identifying predictive biomarkers for immunotherapy response is critical. Given its non‐invasive nature, the gut microbiota holds promise as a potential biomarker for predicting immunotherapy efficacy (Table 1).

**TABLE 1:** Studies on the predictive role of gut microbiota in response to HCC immunotherapy.

Study (year)	Diseases	ICIs	Microbial species	Metabolites	Methods	Predictive outcomes
Zheng et al. (2019) [87]	HCC	Camrelizumab	*Akkermansia muciniphila* , Ruminococcaceae spp	Arbohydrate metabolism, methanogenesis	Metagenomic sequencing	ORR↑
Li et al. (2020) [88]	HCC	Anti‐PD‐1	Clostridiales/Ruminococcaceae	Not analyzed	16S rRNA	PFS↑
Mao J et al. (2021) [89]	HCC, BTC	Anti‐PD‐1	Lachnospiraceae bacterium‐GAM79, *Alistipes* sp. Marseille‐P5997	Bacterial reproduction processes (e.g., UDP/UMP biosynthesis, queuosine biosynthesis)	Metagenomic sequencing and taxonomic profiling	PFS↑, OS↑
Chung et al. (2021) [90]	HCC	Nivolumab	*Akkermansia*, Prevotella/Bacteroides ratio	Not analyzed	16S rRNA sequencing	OR↑
Shen (2022) [91]	HCC	Anti‐PD‐1/Anti‐PD‐L1	No difference observed	No significant association identified	16S rRNA sequencing and shotgun whole‐genome sequencing	No correlate with prolonged OS
Wu et al. (2022) [92]	uHCC	Anti‐PD‐1	*Ruminococcus*, *Klebsiella*	Galactaric acid, 3‐methylindole and lenticin	16S rRNA	ORR↑
Lee et al. (2022) [93]	uHCC	Nivolumab, Pembrolizumab	*Lachnoclostridium*, Lachnospiraceae, *Veillonella*	Ursodeoxycholic acid and ursocholic acid	16S rRNA	PFS↑, OS↑
Zhu et al. (2024) [94]	HCC	Anti‐PD‐1/Anti‐PD‐L1	Actinomyces_sp_ICM47, *Senegalimassilia anaerobia*	Galanthaminone	Metagenomic sequencing	PFS↑, OS↑
Fujii et al. (2024) [95]	HCC	Atezolizumab	*Bacteroides coprocola* , *S. anginosus*	5α‐reductase	16S rRNA/qPCR	ORR↑

Abbreviations: BTC: biliary tract cancer; HCC: hepatocellular carcinoma; ICIs: immune checkpoint inhibitors; ORR: overall response rate; OS: overall survival; PFS: progression‐free survival; qPCR: quantitative polymerase chain reaction; UDP/UMP: uridine diphosphate/uridine monophosphate; uHCC: unresectable hepatocellular carcinoma.

Several studies have explored the link between gut microbiota and immunotherapy outcomes. Zheng et al.'s [87] examined the gut microbiota of 8 HCC patients treated with camrelizumab. They found that responders maintained a stable gut microbiota throughout treatment, while non‐responders exhibited a notable increase in Proteobacteria, which became dominant by the 12th week. Responders also displayed greater microbial richness and gene counts, and 20 bacterial species, including *Akkermansia muciniphila* and Ruminococcaceae, were enriched in these patients. These bacteria are known for promoting gut barrier function and reducing immune suppression. In contrast, non‐responders showed a rise in Bacteroidales, associated with shorter progression‐free survival (PFS) [88]. Additionally, an imbalance in the Firmicutes/Bacteroidetes ratio and low Prevotella/Bacteroidetes ratio were linked to non‐response, while *Akkermansia muciniphila* was associated with favorable outcomes [90]. However, Shen et al.'s team found that gut microbiota profiles, whether at baseline or after treatment, were not correlated with the prognosis of HCC patients undergoing ICIs therapy [91].

In another study combining serum metabolite analysis with gut microbiota profiling, Alpha‐D‐glucose was identified as the only significantly different serum metabolite between responders and non‐responders. Ruminococcus, enriched in responders, positively correlated with serum galactarate, while Klebsiella was linked to 3‐methylindole and coumarin [92]. Similarly, Lee et al. [93] reported significant differences in fecal microbiota composition between responders and non‐responders to ICIs. Lachnoclostridium and Ruminococcaceae dominated in responders, while Prevotella 9 was more abundant in non‐responders. The presence of Lachnoclostridium was strongly predictive of longer PFS and overall survival (OS). Moreover, butyrate‐producing bacteria such as Lachnospiraceae bacterium‐GAM79 and Alistipes sp. Marseille‐P5997 were enriched in the clinical benefit group, while Veillonellaceae was associated with poorer outcomes [89]. Japanese study analyzing the gut microbiome of HCC patients treated with atezolizumab/bevacizumab, *Lactobacillus fermentum* and *Streptococcus anginosus* were enriched in HCC patients, while *Bacteroides stercoris* was associated with non‐response and *Bacteroides coprocola* with response. Quantitative polymerase chain reaction analysis revealed reduced levels of 5α‐reductase genes, essential for the synthesis of isoallolithocholic acid, in HCC patients, further supporting the role of gut microbiota in modulating immunotherapy outcomes [95]. These findings further highlight the potential of specific bacterial species as biomarkers for predicting HCC immunotherapy outcomes.

Another comprehensive analysis of the gut bacteria, fungi, and their metabolites in HCC patients undergoing ICIs therapy revealed significant differences in bacterial and metabolite profiles between different response groups, though differences in fungal communities were minimal. This study developed a predictive model based on 18 bacterial species, which effectively predicted immunotherapy response. Additionally, two bacterial species (*Actinomyces* sp. ICM47 and *Senegalimassilia anaerobia*) and one metabolite (galantamine) were identified as biomarkers of survival, with lower levels correlating with longer survival [94].

Despite these promising findings, there remains a lack of reliable, validated biomarkers for predicting the efficacy and prognosis of immunotherapy in HCC. Most identified biomarkers have yet to undergo large‐scale prospective validation, and their specificity and sensitivity remain uncertain. The emerging evidence points to the potential of gut microbiota as a key player in determining immunotherapy response. Moving forward, comprehensive models that integrate gut microbiota with other biomarkers are essential to optimize patient selection, enhance prognosis, and reduce immune‐related adverse effects.

## 6 The Potential of the Gut Microbiome in Modulating Immunotherapy for HCC

**FIGURE 3:**
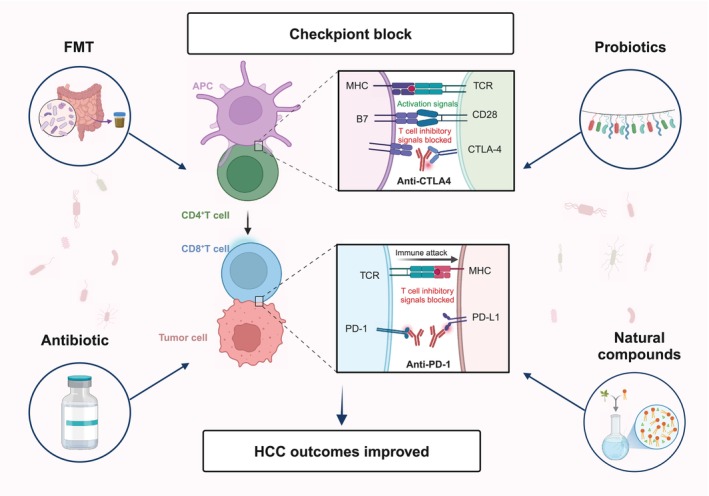
Modulation of gut microbiota enhances HCC immunotherapy efficacy. Various strategies, including probiotics, FMT, antibiotics, and novel natural compounds, can be employed to regulate the gut microbiome. These interventions improve the immune response, thereby boosting the effectiveness of immunotherapy in HCC treatment. APC: antigen‐presenting cell; CTLA‐4: cytotoxic T‐lymphocyte‐associated protein; FMT: fecal microbiota transplantation; MHC: major histocompatibility complex; PD‐1: programmed cell death protein 1; PD‐L1: programmed death‐ligand 1; TCR: T cell receptor.

The gut microbiota plays a pivotal role in modulating the immune response to HCC immunotherapy by influencing both the innate and adaptive immune systems, thereby regulating the efficacy of ICIs. Targeting the gut microbiota offers a novel therapeutic strategy for HCC. Probiotic supplementation, fecal microbiota transplantation (FMT), and antibiotics have shown promise in enhancing HCC immunotherapy outcomes. Moreover, certain novel natural compounds have demonstrated significant potential in augmenting the efficacy of HCC immunotherapy, providing new insights into HCC treatment [96] (Figure 3) (Table 2).

**TABLE 2:** Clinical trials on gut microbiota modulation in HCC immunotherapy.

NCT number	Intervention	ICIs	Study design	*N*	Country	Outcomes	Recruitment status
NCT05690048 [97]	FMT/Vancomycin	Atezolizumab	Interventional, randomized, placebo‐controlled, single blind phase II‐trial	48	Germany	CD8^+^T cell infiltration, AEs, PFS, OS, change of hepatic function	Recruiting
NCT05032014 [98]	*Lactobacillus rhamnosus* Probio‐M9	Anti‐PD‐1	Interventional, randomized, placebo‐controlled, quadruple‐blind	46	China	ORR, PFS, OS	Completed
NCT05750030 [99]	FMT	Atezolizumab, Bevacizumab	Phase IIa, single‐center, open‐label pilot study	12	Austria	AEs, best radiological response, ORR, DCR, PFS, OS	Recruiting
NCT03785210 [100]	Vancomycin	Nivolumab	Single group assignment	22	United States	BOR, AEs, OS	Completed
NCT06551272 [101]	EXL01 (contains an unmodified single strain of *F. prausnitzii* )	Atezolizumab, Bevacizumab	Single group assignment, open label, phase II‐trial	34	France	BOR, AEs, ORR, DCR, PFS, OS	Recruiting
NCT05620004 [102]	*Bifidobacterium bifidum*	Carrilizumab	Randomized, parallel assignment, open label	30	China	ORR, DCR, PFS, OS, AEs	Recruiting

Abbreviations: AEs: adverse events; BOR: best overall response; DCR: disease control rate; FMT: fecal microbiota transplantation; ICIs: immune checkpoint inhibitors; NCT: National Clinical Trial Identifier Number; ORR: objective response rate; OS: overall survival; PD‐1: programmed cell death‐1; PD‐L1: programmed death ligand‐1; PFS: progression‐free survival.

### 6.1 FMT

FMT is a therapeutic method in which the gut microbiome from a healthy donor is transferred to a patient suffering from a particular disease, with the aim of adjusting the microbial community to alleviate disease symptoms [103]. While FMT is primarily used to treat gastrointestinal diseases, it may also inhibit tumor progression by modulating the gut microbiota and reducing the production of certain cytotoxic metabolites. FMT enhances ICIs efficacy by modulating the gut microbiome and promoting anti‐tumor immunity. For example, FMT from ICIs responders can restore gut microbiota diversity and improve ICIs response rates in non‐responders. These effects are mediated by the enrichment of beneficial bacteria (e.g., *Bifidobacterium* and *Enterococcus faecalis* ) and the production of immunomodulatory metabolites, such as SCFAs, which enhance dendritic cell activation and cytotoxic T cell infiltration [104, 105].

In the DEN and high fat diet induced HCC model, FMT from HCC donors increased overall inflammation and T cell responses, but reduced CD8^+^T cells in the liver, thereby weakening the antitumor response. Meanwhile, an increase in CD8^+^PD1^+^T cells was observed in the gut, indicating that gut immune responses may play an important role in shaping the immune microenvironment of HCC. However, FMT from healthy donors enhanced CD8^+^T cell infiltration into the tumor, which may help reactivate CD8^+^T cells and boost their antitumor activity [106].

Although FMT has shown promising results in animal models, its application in human liver cancer treatment still faces significant challenges. Currently, clinical research on the use of FMT in liver cancer treatment is relatively limited, with only 2 phase II trials underway. The FLORA trial (NCT05690048) aims to evaluate the safety and immunogenicity of FMT combined with standard immunotherapy in patients with advanced HCC [97]. Similarly, another phase II trial (NCT05750030) will assess the safety, feasibility, and efficacy of FMT in patients who have responded to PD‐L1 immunotherapy and those who have not responded to atezolizumab/bevacizumab [99]. However, clinical cases directly utilizing FMT for liver cancer treatment remain rare. This may be partly due to the lack of sufficient animal studies to demonstrate the efficacy and safety of FMT in liver cancer therapy. Moreover, there is currently no evidence to suggest that FMT can permanently alter the gut microbiota composition of liver cancer patients. If FMT only temporarily modifies the microbiome, it is crucial to weigh the potential benefits of this short‐term intervention against the risks of fungal, viral, and other pathogenic infections it may introduce, as well as its overall impact on patient outcomes.

### 6.2 Probiotics

Probiotics are a type of microbial supplement that helps maintain the stability of the gut microbiota by introducing one or more beneficial bacteria into the body. In addition to stabilizing the gut microbiome, probiotics can inhibit inflammation, reduce fat accumulation, and prevent the development of gut‐derived endotoxemia [107, 108]. Particularly, specific strains such as *Lactobacillus* and *Bifidobacterium* have shown potential in modulating the gut microbiome and enhancing the efficacy of ICIs. Preclinical studies indicate that these strains can promote anti‐tumor immunity by enhancing dendritic cell activation, increasing cytotoxic T cell infiltration, and reducing immunosuppressive Tregs [109, 110].

In the field of immunotherapy for HCC, the use of probiotics is showing potential in enhancing the efficacy of ICIs. Studies have indicated that probiotics can enhance anti‐tumor immune responses by modulating immune regulatory molecules on the surface of T cells, thereby improving the outcomes of immunotherapy. For instance, a clinical trial involving 46 HCC patients (NCT05032014) is currently recruiting participants to evaluate the impact of the probiotic *Lactobacillus rhamnosus* Probio‐M9 on the response to PD‐1 inhibitor therapy [98]. *L. rhamnosus* has been shown to synergize with immune checkpoint blockade, enhancing the anti‐tumor activity of PD‐1 immunotherapy by increasing tumor infiltration of dendritic cells and T cells [111]. Another clinical trial (NCT05620004) is exploring the potential of *Bifidobacterium* as an anti‐tumor immunomodulator [102]. *Bifidobacterium* can promote dendritic cell maturation and increase the number of CD8^+^T cells, restoring the anti‐tumor effect of PD‐L1 blockade. It can also mediate innate immunity by secreting the metabolite hippuric acid, which suppresses PD‐1 expression and activates NK cells through perforin and IFN‐γ, contributing to the immunogenic remodeling of the TME [109]. Additionally, the probiotic EXL01, containing an unmodified strain of *Faecalibacterium prausnitzii* , is being evaluated in a clinical trial (NCT06551272) for its impact on PD‐1 inhibitor efficacy [101]. *F. prausnitzii* has been shown to increase gut microbial α‐diversity and modulate the composition of the microbiota, enhancing the abundance of beneficial bacteria while reducing potential pathogens [112]. In tumor‐bearing mouse models, *F. prausnitzii* has been found to strengthen ICIs‐induced anti‐tumor immunity and alleviate ICIs‐related colitis [113].

### 6.3 Antibiotic

The impact of antibiotic use on ICIs therapy remains a controversial topic. Some studies suggest that antibiotics may weaken the efficacy of ICIs. For example, a retrospective analysis of 395 patients with advanced HCC treated with ICIs found that the use of antibiotics within 30 days after immunotherapy was associated with higher cancer‐related mortality and all‐cause mortality [114]. Notably, the effect of antibiotics targeting aerobic bacteria was more significant than that of those targeting anaerobic bacteria. Another study showed that in HCC patients receiving nivolumab as a second‐line treatment, those who used antibiotics had a significantly shorter median OS compared to those who did not (5.5 vs. 20 months). Furthermore, a higher percentage of patients who did not receive antibiotics achieved partial or complete response compared to those who did (21% vs. 15%) [115]. However, some studies have not found a negative impact of antibiotics on ICIs efficacy. For instance, an international cohort study involving 450 patients showed that, regardless of disease type or treatment characteristics, the use of antibiotics before or within 30 days of ICIs treatment did not compromise the therapeutic outcomes of immunotherapy in HCC patients [116]. Additionally, a systematic review and meta‐analysis did not show that antibiotic use had a significant impact on OS or PFS in HCC patients receiving ICIs therapy [117]. The varying effects of antibiotics on ICIs efficacy may be related to factors such as the type of antibiotic, cumulative dose, duration of exposure, and route of administration. Some studies have indicated that the use of broad‐spectrum antibiotics, increased cumulative dose, and pre‐treatment antibiotic use are associated with poorer prognosis, whereas antibiotic use in the later stages of treatment does not seem to significantly affect survival outcomes.

Notably, an ongoing clinical trial (NCT03785210) is evaluating whether vancomycin can improve the efficacy of nivolumab in patients with refractory primary liver cancer or liver‐dominant metastatic cancer [100]. Research by Ma et al. found that primary BAs in the gut promote the expression of CXCL16 and the recruitment of NKT cells in the liver. The use of antibiotics such as vancomycin to eliminate Gram‐positive bacteria can reduce the conversion of primary BAs into secondary BAs, thereby increasing the number of NKT cells in the liver and inhibiting liver tumor growth [72]. Overall, the role of antibiotics in ICI therapy remains unclear, and further research is needed to elucidate the underlying mechanisms and optimal management strategies.

### 6.4 Natural Compounds

Natural compounds are gaining increasing attention in the treatment of HCC, particularly in combination with ICIs. By modulating the gut microbiota, these compounds can influence the development and progression of HCC and may enhance the therapeutic efficacy of ICIs.

Natural compounds exert their effects through multiple mechanisms, including optimizing gut microbiota composition and promoting the protection of the intestinal mucosal barrier. The gut microbiota's metabolic conversion of these compounds can significantly enhance their bioavailability, particularly in the case of phytoestrogens such as flavonoids and lignans, which have been extensively studied for their role in modulating the gut microbiota [118, 119]. In terms of immune modulation, natural compounds can enhance antitumor immune responses by activating immune cells and inhibiting the activity of immunosuppressive cells. For example, traditional Chinese medicine components such as safflower yellow can regulate the abundance of gut microbiota, promote collagen degradation in hepatocytes, increase immune cell infiltration in the liver, and optimize the TME in HCC [120]. Additionally, certain natural compounds can reshape the gut microbiota and metabolic networks. For instance, NpRg3 has been shown to reduce levels of 3‐indolepropionic acid and urea in the gut while increasing free fatty acid levels, thereby influencing gut microbial metabolism during HCC treatment and enhancing therapeutic efficacy [121]. The combination therapy of quercetin and anti‐PD‐1 antibody is another example, which modulates gut microbiota, influences TAMs immune responses, and reshapes the TME in HCC. This combined treatment reduces gut microbiota dysbiosis, increases the abundance of Firmicutes, Actinobacteria, and Verrucomicrobia at the phylum level, and enhances the abundance of Dubosiella and Akkermansia at the genus level, while improving macrophage immune function. Specifically, it enhances the expression of CD8a, CD4, and CD11b, as well as the levels of IL‐10 and IFN‐γ, while reducing the expression of interleukin‐4 and IL‐6. Moreover, it modulates the expression of genes associated with M1 and M2 TAMs, thereby improving immune responses within the TME [122].

Although preliminary studies suggest that natural products show great potential in combination therapies for HCC, these findings are largely based on animal experiments or early‐stage research. Further clinical trials are necessary to validate their safety and efficacy. Future research should focus on how to effectively translate these natural products into clinical practice to ensure their optimal effectiveness in HCC treatment.

## 7 Challenges in Translating Microbiome Biomarkers

Despite the growing interest in microbiome research and its potential applications in HCC, translating microbiome findings into clinically validated biomarkers and therapies faces significant challenges. First, inter‐individual variability in gut microbiota composition across different populations complicates the identification of universal biomarkers. Factors such as genetics [123], age [124], geography [125], and lifestyle [54] contribute to this variability, making it difficult to develop biomarkers that are applicable to all HCC patients. Second, technical inconsistencies in sequencing methodologies, including differences in sample collection, DNA extraction, sequencing platforms (e.g., 16S rRNA vs. shotgun metagenomics), and bioinformatics pipelines, can lead to conflicting results [126]. Standardized protocols and cross‐laboratory validation are essential to ensure reproducibility and comparability across studies [127]. Finally, confounding factors, such as diet [128], antibiotic use [129], and underlying liver disease, significantly influence the gut microbiota and its metabolic output. These factors must be carefully controlled in clinical studies to isolate the effects of specific microbial changes on HCC progression and treatment outcomes. Addressing these challenges will require a multidisciplinary approach, including large‐scale longitudinal studies, advanced computational tools for data integration, and the development of standardized protocols for sample collection and analysis. Additionally, integrating multi‐omics data (e.g., metagenomics, metabolomics, and proteomics) may provide a more comprehensive understanding of the gut‐liver axis in HCC and facilitate the identification of robust biomarkers.

## 8 Conclusions

The gut‐liver axis presents a unique physiological link, highlighting the critical role of gut microbiota in the development and progression of HCC, particularly through metabolic regulation and immune modulation. The bidirectional relationship between gut microbiota and HCC offers promising avenues for early treatment. Targeting the gut microbiome may emerge as an innovative strategy for preventing and treating HCC. Future research should prioritize translating these findings into clinical applications, focusing on elucidating the interactions between gut microbiota, HCC, and therapeutic interventions to optimize personalized treatment strategies.

## Author Contributions

Kunmin Xiao and Kexin Li: writing – original draft. Kunlin Xiao: visualization. Jinzu Yang and Lei Zhou: writing – review and editing, conceptualization. All authors contributed to this work and approved the final manuscript.

## Ethics Statement

The authors have nothing to report.

## Conflicts of Interest

The authors declare no conflicts of interest.

## Data Availability

No data were used for the research described in the article.
